# Preventive treatment patterns in the adult migraine population: an observational UK study over 7 years

**DOI:** 10.1186/s12875-023-02242-y

**Published:** 2024-01-24

**Authors:** David Kernick, Nazanin Kondori, Andrew Pain, Julie Mount, Camilla Appel, Michael Ranopa, Tania Gulati

**Affiliations:** 1 St Thomas Health Centre, Exeter, UK; 2 Eli Lilly and Company Ltd, Lilly House, Basing View, Basingstoke, Hampshire RG21 4FA UK

**Keywords:** Calcitonin gene-related peptide antagonists, CPRD, Migraine epidemiology, Migraine preventive medications

## Abstract

**Background:**

Calcitonin gene-related peptide monoclonal antibodies (CGRP mAbs) are recommended by the United Kingdom National Institute of Health and Care Excellence for the prevention of migraine as treatment beyond third line. We report migraine prevalence and preventive treatment patterns in the adult United Kingdom primary care population over a 7.5-year period, focusing on patients ceasing ≥ 3 oral preventive medication classes.

**Methods:**

Study populations were retrieved from the Clinical Practice Research Datalink GOLD database (study period: 19 September 2012 to 1 January 2020; inclusion criteria: ≥12 months follow-up, current-in-dataset, adult on 1 January 2020). Patients who used ≥ 1 oral preventive medication with ≥ 3-year follow-up after first prescription were considered preventive treatment users; class cessation was defined as cessation without evidence of restart within 6 months from end-of-supply date.

**Results:**

On 1 January 2020, 3.0% of the total study population were diagnosed with migraine (n = 81,190/2,664,306); of these, 42.4% were preventive treatment users (n = 34,448/81,190). The most frequently used oral migraine preventive medication classes were beta-blockers (n = 14,713), tricyclic antidepressants (n = 14,415) and antiepileptics (n = 6497). Among preventive treatment users, 7.7% (n = 2653/34,448) ceased ≥ 3 oral preventive medication classes; of these, 21.7% (n = 576/2653) had been referred to a neurologist.

**Conclusions:**

Compared to existing population-based estimates of migraine prevalence, our data further corroborates that a considerable proportion of patients with migraine do not seek treatment. Among those who sought primary care within a 7.5-year period, almost half received empirical oral preventive treatment. Importantly, nearly 1 of 10 preventive treatment users ceased ≥ 3 oral preventive medication classes, highlighting a need for additional therapeutic options. These patients may benefit from CGRP antagonists and/or injectable onabotulinumtoxinA; however, only a minority was referred to specialist care, where these options would be more available.

**Trial Registration:**

Not applicable.

**Supplementary Information:**

The online version contains supplementary material available at 10.1186/s12875-023-02242-y.

## Background

Although migraine is a highly prevalent disease associated with a notable level of disability, [1, 2] many people with migraine do not seek help and when they do their needs are not well addressed [3, 4]. Diagnosis is often incorrect and treatment suboptimal [5–8]. In addition, adherence and persistence of oral migraine preventive medications are poor due to lack of efficacy and/or low tolerability [9].

Until recently, therapeutic choices for migraine preventive treatment were drugs originally developed for indications other than migraine, such as antihypertensives, antidepressants, antiepileptics and onabotulinumtoxinA [10]. The advent of calcitonin gene-related peptide monoclonal antibodies (CGRP mAbs) appears to rapidly change the field, being a targeted treatment against a pathway that is implicated in the pathophysiology of the disease. In addition, CGRP mAbs are associated with a favourable safety profile [11, 12].

In view of randomized controlled trials (RCTs) and real-world evidence which have expanded the evidence and knowledge for those treatments, the European Headache Federation (EHF) has recently published an update of 2019 guidelines on the use of those treatments, recommending that they should be included as a first-line treatment option in individuals with migraine who require preventive treatment [13, 14]. That said, the United Kingdom National Institute of Health and Care Excellence (UK NICE) recommends CGRP mAbs for fourth-line treatment and beyond.

In the UK, there is a current shift to healthcare being delivered from ‘integrated care systems’, which focus on removing the traditional divisions between hospitals and general practice [15]. A natural extension of this development will be that integrated budgets shall be allocated for specific disease areas with outcome-based contracts. This development offers major opportunities to improve migraine care through service redesign. However, for new services to be delivered effectively, clinicians and healthcare commissioners need data about current primary care epidemiology, secondary care referral patterns and the adoption of existing management guidelines.

Given the lack of current epidemiological and preventive treatment pattern information on migraine in the UK, the aim of this article was to provide up-to-date primary care epidemiological data to inform the development of migraine services by analysing a large UK general practitioner database. Study objectives were: (a) to describe the prevalence of migraine consultation in an adult UK general practice population; (b) to describe referral patterns to secondary care; (c) to analyse patterns of oral preventive treatments and whether current guidelines are followed; and (d) to estimate the potential demand for recommended fourth-line preventive drugs following failure of current UK NICE guidelines.

## Methods

### Ethics and consent to participate

The study was set within the UK Clinical Practice Research Datalink (CPRD) GOLD database [16, 17]. The CPRD GOLD database contains data from over 20 million patients registered in nearly 1000 general practices [18]. CPRD patients are broadly comparable to the UK general population in terms of age, sex and ethnicity [19]. Diagnoses are recorded using read codes (a coding system for clinical terms used in the UK National Health System since 1985) [20].

Data from the CPRD were obtained under licence from the UK Medicines and Healthcare products Regulatory Agency. The use of CPRD data for this study was approved through the CPRD’s Research Data Governance Process (eRAP protocol 2021_000567, approved 24 November 2021). As this study was purely observational, Expert’s Review Committee falls under the annual ethics application that CPRD routinely completes. As CPRD data are anonymized, informed consent was not required for use in the study [16].

### Study design and study population

The study period was defined as 19 September 2012 to 1 January 2020. The start date was set to correspond to the date the first NICE Clinical Guideline on ‘Headache in over 12s: diagnosis and management’ was published [21]. Although the study could have been extended beyond January 2020, it was judged appropriate to end it prior to the onset of the COVID-19 pandemic in order to avoid any potential effects on primary care access.

**Fig. 1:**
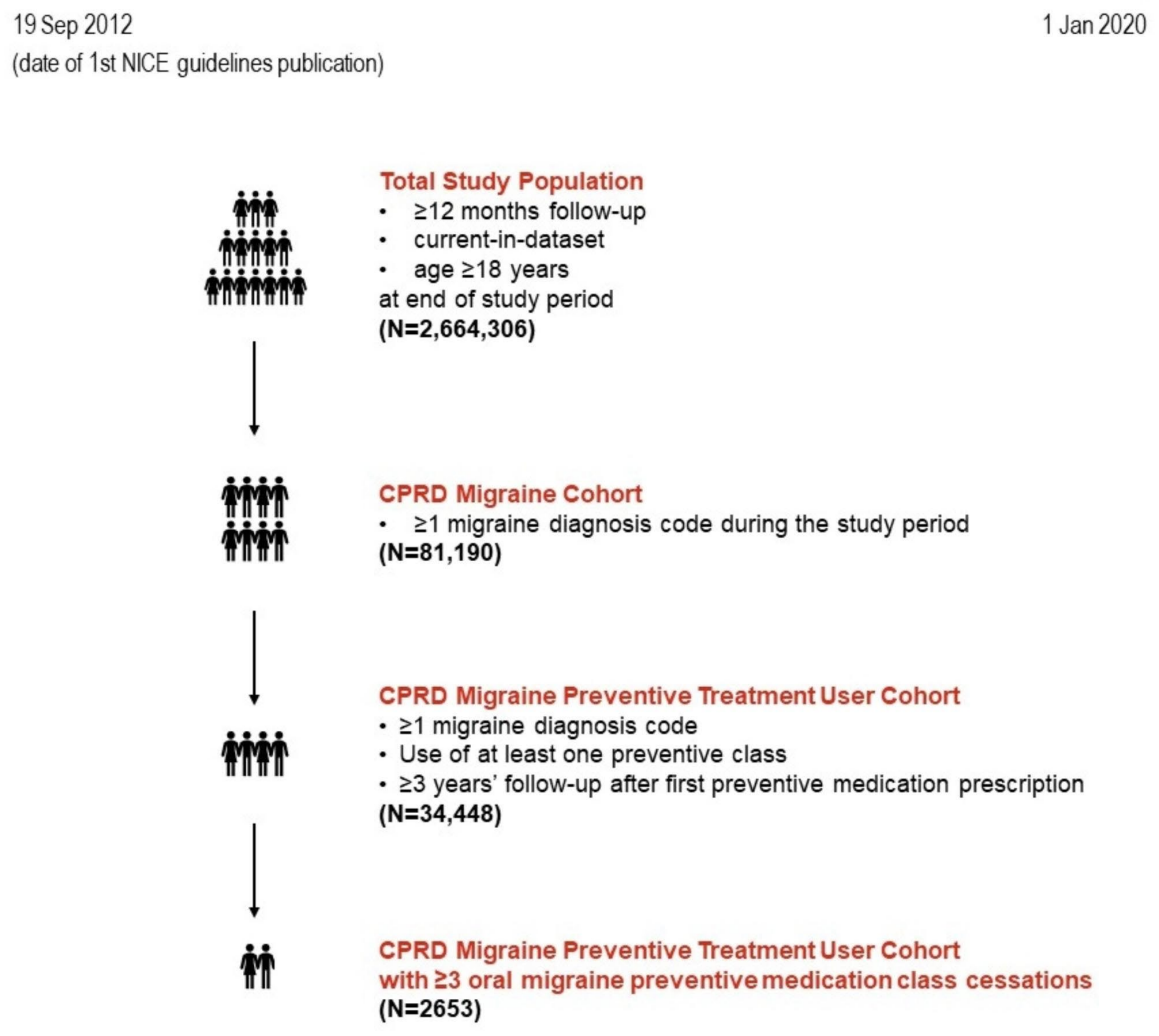
Study period and study cohorts. CPRD: Clinical Practice Research Datalink; N: number of patients in total population; NICE: National Institute for Care and Excellence

The total study population was extracted from the CPRD population based on the following criteria: ≥12 months registered in the Practice with up-to-standard data, alive and aged ≥ 18 at the end of the study period (1 January 2020) (Fig. 1). This population served as a denominator for estimating the prevalence of migraine in the adult primary care population on 1 January 2020 (Supplemental Table 1).

From the total population we identified further three sub-populations (Fig. 1): (a) the CPRD Migraine Cohort, which consisted of individuals within the total study population who had a record of ≥ 1 migraine diagnosis read code during the study period (Supplemental Table 2); (b) the CPRD Migraine Preventive Treatment User Cohort, which consisted of CPRD Migraine Cohort members who received ≥ 1 prescription of an oral preventive medication during the study period and had ≥ 3-year follow-up after the first oral preventive medication prescription (Supplemental Table2); and, (c) the CPRD Migraine Preventive Treatment User Cohort with ≥ 3 oral migraine preventive medication class cessations. For patients with migraine using preventive medication (CPRD Migraine Preventive Treatment User Cohort), we intentionally selected those with a long follow-up time (≥ 3 years) after first prescription of an oral preventive medication during the study period, in order to evaluate treatment cessations, switches to next line of treatment and referrals to secondary care within an extended time frame.

The CPRD Migraine Preventive Treatment User Cohort was used for longitudinal assessment of oral preventive treatment use during the medication assessment period; the index date for the preventive medication assessment period was the date of the first oral preventive medication prescription during the study period and follow-up ended on 1 January 2020. Data were collected for both unique (specific) medications, as well as unique medication classes. The number of unique oral preventive medications/medication classes prescribed, the time on unique medications/medication classes and the number of lines of therapy were counted. Medication cessation was defined as the end of supply of medication plus 60 days AND no evidence of restart of the same class within 6 months of end of supply date. (If the end of study period (1 January 2020) was prior to the end of supply date plus 6 months, this was counted as a cessation). The CPRD Migraine Preventive Treatment User Cohort with ≥ 3 oral migraine preventive medication class cessations was used to estimate the potential demand in terms of prevalent patients for fourth-line preventive agents.

History of ≥ 1 referral to secondary care (neurologist) during the study period was assessed for all three sub-populations.

### Statistical analysis

Prevalence is reported over the study period as n/1000 (95% confidence interval (CI)) for the overall population and by age group (18 − 65 and > 65 years). Descriptive statistics for patient characteristics were summarized for the CPRD Migraine Cohort, the CPRD Migraine Preventive Treatment User Cohort and the CPRD Migraine Preventive Treatment User Cohort with ≥ 3 oral migraine preventive medication class cessations. Time on unique medications/medication classes was summarised as the mean (SD) and the median number of days on each unique preventive therapy. Missing values were not imputed, with the exception that where the days supplied variable for a medication was missing but evidence of a prescription was available. In this case, the days supplied variable was set to 1 day.

## Results

### Characteristics of adult patients with migraine in UK primary care across all study cohorts

A total of 2,664,306 patients fulfilled the criteria to be included in the total study population, as described in the Methods section (Supplemental Table 1). This dataset represented adults who had sought primary care and served as a denominator for estimating the prevalence of migraine (on 1 January 2020) in the adult primary care population rather than in the total population. Among these patients, 81,190 had ≥ 1 migraine read code (designated the CPRD Migraine Cohort, 91.2% of whom were aged 18–65 years). Of these, 60% (n = 48,704) had used ≥ 1 oral preventive class within the study period. Patients who had used ≥ 1 oral preventive class within the study period and also had ≥ 3 years follow-up after the first oral preventive medication formed the CPRD Migraine Preventive Treatment User Cohort [42.4% (of the CPRD Migraine Cohort, n = 34,448]. Among the latter, 7.7% (n = 2653) had ceased ≥ 3 oral preventive medication classes, forming the CPRD Migraine Preventive Treatment User Cohort with ≥ 3 oral migraine preventive medication class cessations (Table 1).

**Table 1:** Patient characteristics across study cohorts

	CPRD Migraine Cohort (N = 81,190)	CPRD Migraine Preventive Treatment User Cohort (N = 34,448)	CPRD Migraine Preventive Treatment User Cohort with ≥ 3 oral migraine preventive medication class cessations^*^ (N = 2653)
Mean age (years, SD)	42.5 (15.5)	45.8 (15.7)	45.0 (14.6)
18–65 years > 65 years	74,051 (91.2) 7139 (8.8)	30,443 (88.4) 4005 (11.6)	2410 (90.8) 243 (9.2)
Gender – female (n, %)	62,754 (77.3)	27,226 (79.0)	2200 (82.9)
History of referral to neurologist (n, %)	5715 (7.0)	3801 (11.0)	576 (21.7)

CPRD: Clinical Practice Research Datalink; N: number of patients in total population; n: number of patients in group

*Medication class was considered ceased when no evidence of restart of the same medication class within 6 months from end-of-supply date during the medication assessment period

For the CPRD Migraine Preventive Treatment User Cohort, the index date for the preventive medication assessment period was the index preventive therapy prescription date during the study period and the end date was 1 January 2020.

Most patients were female (77.3% within the CPRD Migraine Cohort, 79.0% within the CPRD Migraine Preventive Treatment User Cohort and 82.9% within the CPRD Migraine Preventive Treatment User Cohort with ≥ 3 oral migraine preventive medication class cessations). The mean age within each cohort was 42.5 (SD 15.5), 45.8 (SD 15.7) and 45.0 (SD 14.6) years, respectively. Of note, only 21.7% of the patients with ≥ 3 preventive medication class cessations had a history of referral to a neurologist by their general practitioner (GP) during the study period (Table 1).

### Prevalence of adult patients with migraine who sought primary care

Among the 81,190 patients identified with ≥ 1 migraine read code (CPRD Migraine Cohort) during the 7.5-year study period, the number with migraine, calculated on 1 January 2020, was 30.5/1000 (95% CI 30.3–30.7), and was higher in patients aged 18–65 years (35.9/1000, 95% CI 35.6–36.1) than in those aged > 65 years (11.9/1000, 95% CI 11.6–12.2) (Supplemental Table 3).

### Patients with ≥ 3 oral migraine preventive medication class cessations

Overall, the number of patients with migraine who had ceased ≥ 3 oral preventive medication classes on 1 January 2020 was 1.0/1000 (95% CI 0.96–1.03). This number was highest in the 18- to 65-year age group (1.17/1000, 95% CI 1.12–1.21) (Supplemental Table 4).

### Preventive treatment patterns

**Fig. 2:**
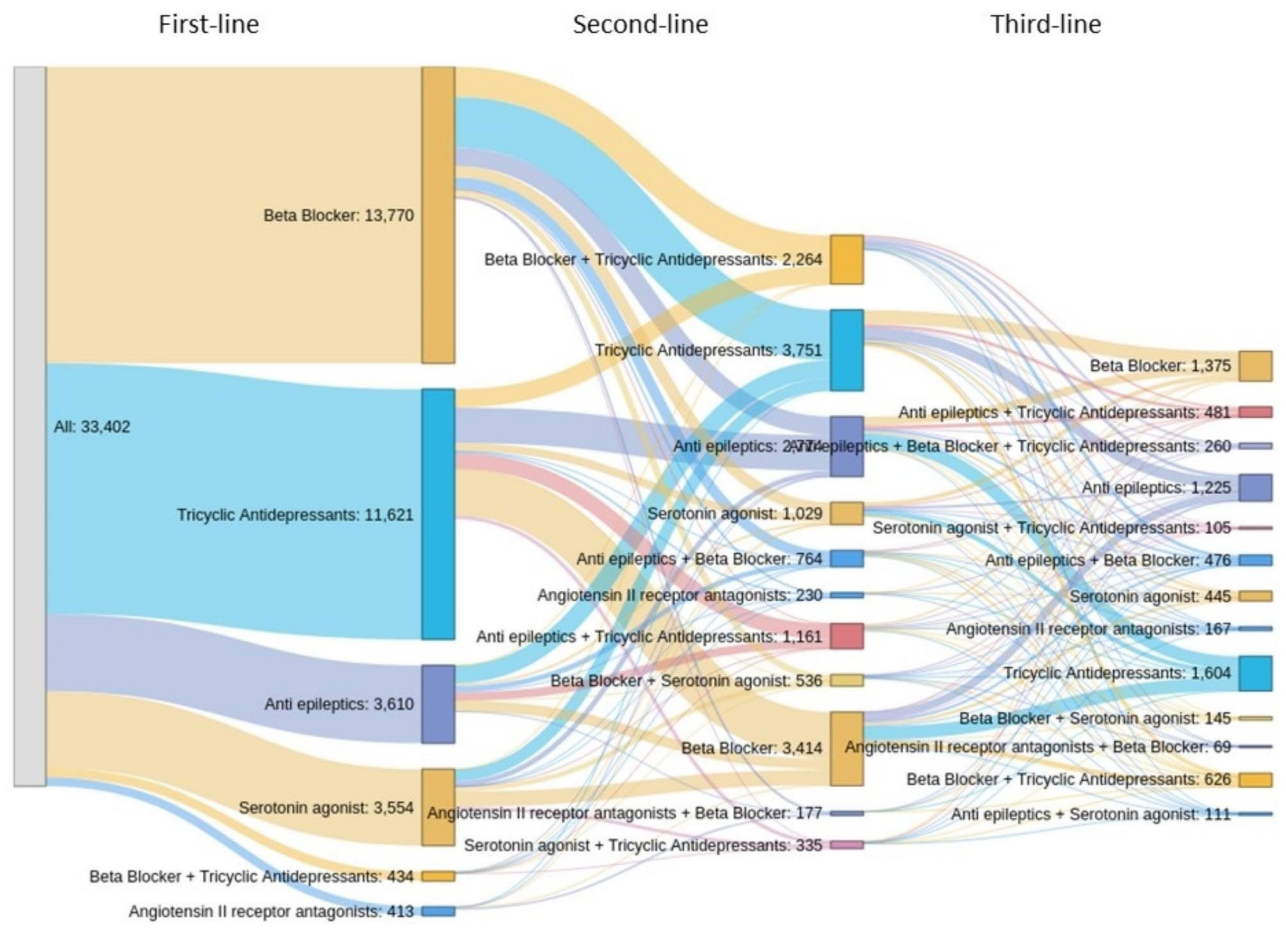
Oral preventive medication class regimens by line of therapy – CPRD Migraine Preventive Treatment User Cohort (N = 34,448)^a^. ^a^Treatments that comprise < 1% are not displayed, so the patient count is lower than the cohort size. CPRD: Clinical Practice Research Datalink; N: number of patients in total population. Clarification: (i) In the second column, third block from the top “Anti epileptics: 2,774” is stated, and (ii) in the third column, third block from the top “Anti epileptics + Beta Blocker + Tricyclic Antidepressants: 260” is stated

Within the CPRD Migraine Preventive Treatment User Cohort (n = 34,448), the median number of unique medication class use during the preventive medication assessment period was 2 (range 1–6). The most frequently used oral migraine preventive medication classes were beta-blockers (n = 14,713) and tricyclic antidepressants (n = 14,415), followed by antiepileptics (n = 6497) and serotonin antagonists (n = 4623). For these, the median time on treatment was 80, 65, 145 and 64 days, respectively; of note, the time on each preventive medication was quite variable among preventive treatment users, as shown by the difference between median and mean values and the high SD (Table 2) (Fig. 2).

**Table 2:** Time on unique medication classes within the CPRD Migraine Preventive Treatment User Cohort (N = 34,448)

Medication class	N (with at least one prescription)	Median time on treatment (days)*	Mean time on treatment (days (SD))*
Tricyclic antidepressant	14,415	65	281 (448)
Calcium channel blocker	327	84	321 (470)
Serotonin agonist	4,623	64	246 (400)
Beta-blocker	14,713	80	298 (467)
Anti-epileptic	6,497	145	355 (465)
Angiotensin II receptor antagonist	714	134	363 (512)

CPRD: Clinical Practice Research Datalink; N: number of patients in total population

* Time on treatment was assessed during the preventive medication/medication class assessment period

**Fig. 3:**
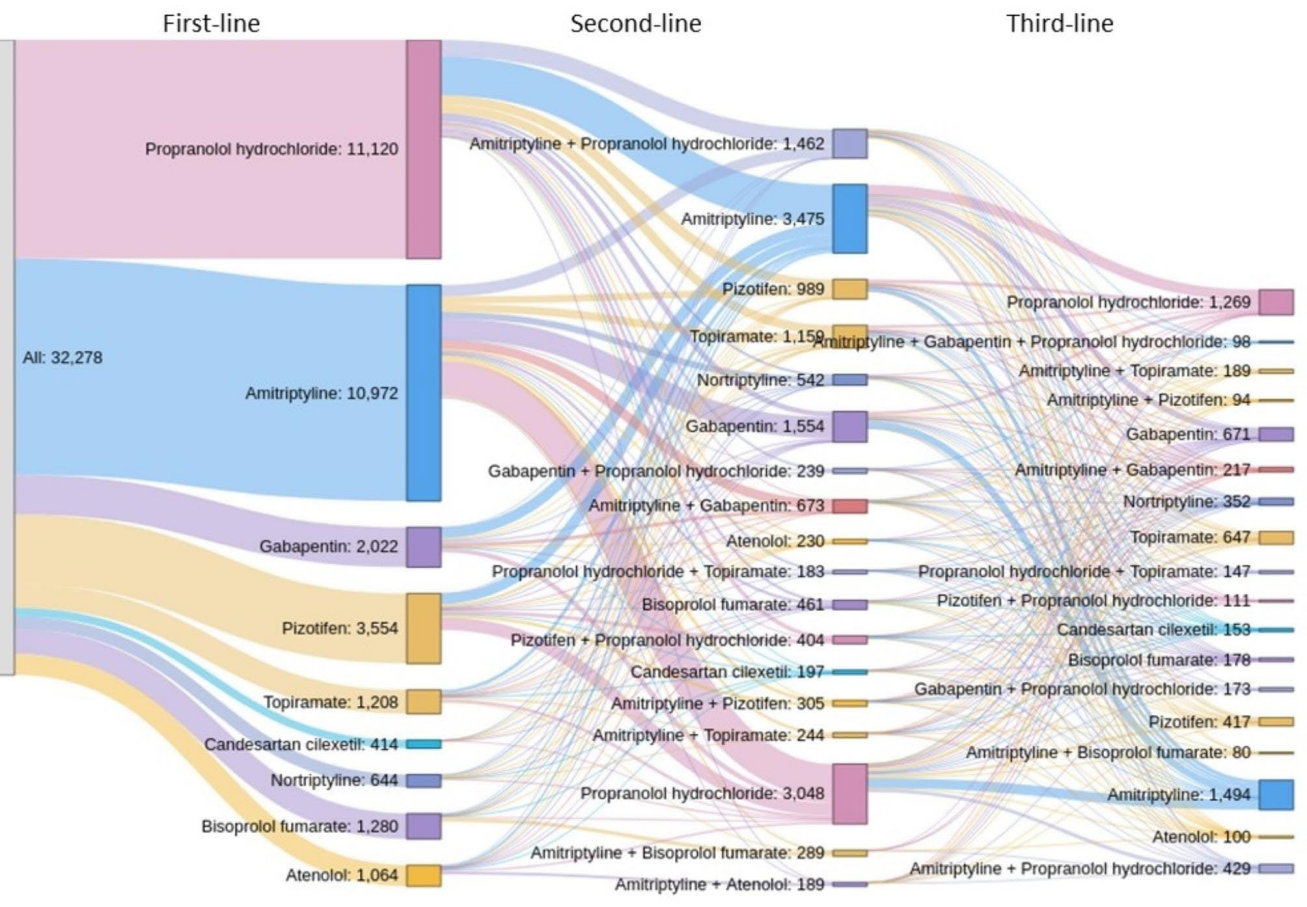
Oral preventive medication regimens by line of therapy – CPRD Migraine Preventive Treatment User Cohort (N = 34,448)^a^. ^a^Treatments that comprise < 1% are not displayed, so the patient count is lower than the cohort size. CPRD: Clinical Practice Research Datalink; N: number of patients in total population. Clarification: (i) In the second column, fourth block from the top “Topiramate: 1,159” is stated, and (ii) in the third column, second block from the top “Amitriptyline + Gabapentin + Propranolol hydrochloride: 98” is stated.

In terms of unique preventive medications (instead of medication classes), the most common were amitriptyline (n = 13,752) and propranolol hydrochloride (n = 13,087), followed by pizotifen (n = 4623) and gabapentin (n = 4169). For these, the median time on treatment was 56, 62, 64 and 106 days, respectively, again quite a variation within each class. Topiramate was less frequently used (n = 2555 individuals overall; median time on treatment 154 days) (Table 3); it ranked sixth as first-line medication (n = 1208), following propranolol hydrochloride (n = 11,120), amitriptyline (n = 10,972), pizotifen (n = 3554), gabapentin (n = 2022) and bisoprolol fumarate (n = 1280) (Fig. 3).

**Table 3:** Time on unique medications within the CPRD Migraine Preventive Treatment User Cohort (N = 34,448)

Medication	N (with at least one prescription)	Median time on treatment (days)*	Mean time on treatment (days (SD))*
Amitriptyline	13,752	56	267 (438)
Atenolol	1133	161	490 (629)
Bisoprolol fumarate	1326	168.5	424 (551)
Candesartan cilexetil	714	134	363 (512)
Flunarizine	14	58	155 (254)
Gabapentin	4169	106	301 (432)
Metoprolol tartrate	173	121	428 (630)
Nadolol	18	187.5	316 (344)
Nortriptyline	1485	94	253 (390)
Pizotifen	4623	64	246 (400)
Propranolol hydrochloride	13,087	62	262 (423)
Sodium valproate/valproic acid	617	161	413 (546)
Timolol maleate	104	379	641 (696)
Topiramate	2555	154	345 (438)
Verapamil	314	87	327 (476)

CPRD: Clinical Practice Research Datalink; N: number of patients in total population

*Time on treatment was assessed during the preventive medication/medication class assessment period

## Discussion

This study, covering an approximately 7.5-year period, found the prevalence of migraine in the UK adult population who sought care by a GP to be 3.0% on 1 January 2020. Compared to population estimates of 14.3%, 22 it supports the observation that over 50% of people with migraine never consult a doctor despite experiencing severe migraine-related disability [3, 4, 23]. Almost half (42%) of patients with migraine who did consult a GP received an oral preventive medication. Approximately 1 out of 10 preventive treatment users (7.7%) ceased ≥ 3 medication classes, pointing to an unmet therapeutic need. Even so, only 1 of 5 preventive treatment users who ceased ≥ 3 medication classes (21.7%) were referred to secondary care, where CGRP antagonists and/or onabotulinumtoxinA would be available.

Not only is the actual population prevalence expected to be quite a bit higher, but it is possible that our result also underestimates the true migraine prevalence among GP consultations. First, migraine might be underdiagnosed by GPs [6]. Second, it cannot be excluded that patients with migraine may have been missed from the study population due to diagnosis through a read code not included in the study code list. In addition, it is also possible that people with migraine have visited a GP for other health-related problems which they deemed more significant and did not report migraine symptoms.

In our study, propranolol hydrochloride and amitriptyline were the most frequently used oral first-line preventive medications, in line with NICE guidelines [21]. Topiramate on the other hand, although a NICE recommended first-, second- and third-line preventive treatment option, was less frequently used [21]. Conversely, gabapentin was one of the most frequently used medications in first-line treatment, in contrast to NICE guidelines which specifically discourage its use as a preventive migraine treatment.

Reports of the proportion of people with migraine stopping or switching first-line medication reach up to 90% [9, 24–27]. Also, it is estimated that approximately one-third of migraine preventive treatment users will have stopped treatment altogether at the end of 1 year – the most common reason being adverse events and lack of efficacy [9, 27, 28] A recent survey conducted by the UK Migraine Trust including > 1800 people with migraine across the UK reports that less than one-third of respondents (32%) are satisfied with the care and treatment they receive. In this survey, only 15% of respondents believe that the NHS is able to manage migraine well; at the same time, 28% of respondents have ended up paying to see a health professional privately about their migraine in the last 5 years [29]. In our study, the median length of stay on a medication class was at least 2 months, which would generally be perceived as an adequate length of time for evaluating actual benefit. The median number of prescribed medication classes among preventive treatment users was 2, indicating that for most of them first-line treatment was most likely either ineffective or not tolerated. Of course, it cannot be excluded that oral migraine preventive medications could have been prescribed for other indications in migraine patients. For example, beta-blockers, calcium channel blockers and angiotensin II receptor antagonists are also being prescribed for arterial hypertension; antidepressants are being prescribed for mood disorders, which are in fact common among patients with migraine [30]. However, in this study, the population with a migraine diagnosis and a record of ≥ 3 oral migraine preventive medication class cessations during the study period most likely represents patients with migraine in whom multiple migraine preventive medications have failed. The Burden of Episodic and Chronic Migraine in Europe (BECOME) study in 2021 reported an even higher proportion (15.3%) of patients with migraine experiencing ≥ 4 preventive treatment failure(s), possibly because data were captured solely from tertiary centres [31].

NICE currently recommends either onabotulinumtoxinA (Botox) for chronic migraine or CGRP antagonists for episodic or chronic migraine when three recommended preventive agents have failed [32–35]. Based on the 7.5-year period prevalence data from this study, and assuming that all oral preventive medication class cessations at and beyond third-line were due to response failure and/or adverse event, we can estimate that 2653 patients were eligible for fourth-line therapy at the end of the study period (1 January 2020). Admittedly, this study evaluated only oral migraine preventive medications and did not identify patients who were prescribed onabotulinumtoxinA; however, the latter is mostly administered in a secondary care setting as a preventive treatment for migraine.

## Strengths/Limitations

The study covered an extensive time period of approximately 7.5 years up to 1 January 2020; most recent migraine data (2020−2021) were deliberately excluded in order to avoid potential noise due to the COVID-19 pandemic. The study identified patients with migraine with at least one read code recorded within the study period; thus, cases with a read code before 19 September 2012 that was not repeated within the study period may have been missed. Similarly, referrals to a neurologist that occurred before 19 September 2012 may have been missed. However, given the extensive study period, the number of missed cases for both scenarios is expected to be low. However, it is important to acknowledge that our results may underestimate the true migraine prevalence among GP consultations, either due to underdiagnosing by GPs [6] or diagnosis through a read code not included in the study code list, as mentioned above. It is also important to clarify that our results solely refer to patients with migraine who sought for primary care; thus, population estimates can only be inferred.

Of note, this study was not designed to address the reason(s) for patients not continuing to the next line of treatment. It may be due to (a) patients not returning for review; (b) natural regression of migraine; or (c) GPs electing not to explore preventive medication further. However, the proportion of patients with migraine who ceased ≥ 3 lines of oral migraine preventive medication classes during the study period (7.7% of the CPRD Migraine Preventive Treatment User Cohort) is more likely to represent a group with poor efficacy/tolerability of existing preventive treatments and a need for additional options. For this group of patients, the possibility of error because of migraine preventive medication being prescribed for another indication is also low. However, the study did not address drug compliance or dose regimens. Thus, patients who were not taking their medication appropriately would potentially be classified as treatment failures. The same could be true for women of childbearing potential who might be taken off their oral migraine prophylaxis prior to or during pregnancy and therefore be falsely considered as treatment failure.

Finally, the study could not address disease characteristics (e.g. severity), medication adverse events, medication use for acute treatment, medication overuse, and neither could it assess patient quality of life, healthcare resource utilization metrics or barriers to care. Especially, the social aspect is particularly relevant for migraine; the satisfaction rate for healthcare is globally low, and a recently released UK-based survey by the Migraine Trust reported less than one-third of respondents (32%) being satisfied with the care and treatment they receive, further highlighting the existing unmet clinical need.

## Conclusion

Overall, this study highlights the need for increased awareness among both patients with migraine and primary care physicians in the UK. The development of practical guidelines for patient referral to secondary care may facilitate better access to more effective treatments, including novel targeted therapies. There are some encouraging signs of both a local and national headache treatment pathway, which will hopefully aid the management of headache disorders [36]. The current study may serve as a ‘baseline’ for evaluating these new interventions, as they were not implemented during the study period.

### Electronic supplementary material

Below is the link to the electronic supplementary material.

Supplementary Material 1

## Data Availability

The datasets used and/or analysed during the current study are available from the corresponding author on reasonable request.

## Abbreviations

BECOME: Burden of Episodic and Chronic Migraine in Europe
CGRP: calcitonin gene-related peptide
CI: confidence interval
CPRD: Clinical Practice Research Datalink
GP: general practitioner
NHS: National Health Service
NICE: National Institute for Care and Excellence
SD: standard deviation
UK: United Kingdom
USA: United States of America
