# Supplementary material for: Preventive treatment patterns in the adult migraine population: an observational UK study over 7 years
Source: BMC Prim Care. 2024 Jan 24;25:34. doi: 10.1186/s12875-023-02242-y (PMC10807158; doi:10.1186/s12875-023-02242-y)
Supplement: Supplementary file 1 — Supplementary Material 1 [file 12875_2023_2242_MOESM1_ESM.docx]

Supplemental Table 1. CPRD Prevalence Denominator population on 1 January 2020 (attrition table).

|  | **N** | **% (of row above)** |
| --- | --- | --- |
| CPRD total population^*^ | 20,418,567 | 100 |
| At least 12 months of Registered in Practice (Current registration date before 1 Jan 2019) | 19,817,320 | 97.06 |
| At least 12 months of up to standard data (Up to standard date before 1 Jan 2019) | 19,136,208 | 96.56 |
| No death record prior to 1 Jan 2020 | 17,556,929 | 91.74 |
| No transfer out date prior to 1 Jan 2020 | 7,550,195 | 43.00 |
| No Last collection date prior to 1 Jan 2020 | 3,302,228 | 43.37 |
| Aged ≥18 on 1 Jan 2020 | 2,664,306 | 80.68 |

^*^CPRD GOLD Release Notes 2022^12^

CPRD: Clinical Practice Research Datalink; N: number of patients in total population.

Supplemental Table 2. Read codes and medication list.

Read codes^*^

| **Medcode** | **Read code** | **Read term** |
| --- | --- | --- |
| 161 | F26..00 | Migraine |
| 321.00 | F262500 | Periodic migrainous neuralgia |
| 2424 | F261.00 | Common migraine |
| 3220 | F260.00 | Classical migraine |
| 3658 | F26y000 | Hemiplegic migraine |
| 5029 | 1474.00 | H/O: migraine |
| 5509 | F262.00 | Migraine variants |
| 9004 | F262300 | Basilar migraine |
| 9633 | F261000 | Atypical migraine |
| 10583 | F262400 | Ophthalmic migraine |
| 11138 | K584.11 | Migraine - menstrual |
| 11389 | 8B6N.00 | Migraine prophylaxis |
| 12511 | F26y100 | Ophthalmoplegic migraine |
| 14700 | F26z.00 | Migraine NOS |
| 22685.00 | F26y200 | Status migrainosus |
| 23621 | F262z00 | Migraine variant NOS |
| 27930 | F26y300 | Complicated migraine |
| 28031 | F26y.00 | Other forms of migraine |
| 28092 | F26yz00 | Other forms of migraine NOS |
| 41497 | F261z00 | Common migraine NOS |
| 53813 | Fyu5300 | [X]Other migraine |
| 65262 | F26y111 | Moebius' ophthalmoplegic migraine |
| 103451 | 1474000 | H/O migraine with aura |
| 103502 | F260.11 | Migraine with aura |
| 103602 | F261.11 | Migraine without aura |

^*^Read codes are a coding system of clinical terms used in the UK National Health System (NHS) since 1985 (https://www.data.gov.uk/dataset/f262aa32-9c4e-44f1-99eb-4900deada7a4/uk-read-code).

NOS: not otherwise specified

Preventive treatments and classes

| Migraine preventive medications and classes defined for this study are as follows (class in bold):  **Tricyclic antidepressant:**  Amitriptyline  Nortriptyline  **Calcium channel blocker**:  flunarizine  verapamil  **Serotonin agonist:**  pizotifen  **Beta-blocker**:  atenolol  bisoprolol fumarate  metoprolol tartrate  nadolol  propranolol hydrochloride  timolol maleate  **Anti-epileptics**:  sodium valproate/ valproic acid  gabapentin  topiramate  **Angiotensin II receptor antagonists:**  candesartan cilexetil |
| --- |

**Supplemental Table 3**. Study period prevalence of migraine consultation in the adult UK primary care population.

|  | **n (numerator)** | **N (CPRD prevalence denominator population)** | **Prevalence***  **(n/1000)** | **95% CI** |
| --- | --- | --- | --- | --- |
| Overall | 81,190 | 2,664,306 | 30.5 | 30.3–30.7 |
| Aged 18–65 years | 74,051 | 2,064,486 | 35.9 | 35.6–36.1 |
| Aged >65 years | 7139 | 599,820 | 11.9 | 11.6–12.2 |

CI: confidence interval; CPRD: Clinical Practice Research Datalink, N: number of patients in the total study population; n: number of patients with migraine on 1 January 2020 in the group.

*On 1 January 2020

**Supplemental Table 4.** Study period prevalence of population with ≥3 oral migraine preventive medication class cessations.

|  | **n (numerator)** | **N (CPRD prevalence denominator population)** | **Prevalence (n/1000)*** | **95% CI** |
| --- | --- | --- | --- | --- |
| Overall | 2653 | 2,664,306 | 1.00 | 0.96–1.03 |
| Aged 18-65 years | 2410 | 2,064,486 | 1.17 | 1.12–1.21 |
| Aged >65 years | 243 | 599,820 | 0.40 | 0.36–0.46 |

CI: confidence interval; CPRD: Clinical Practice Research Datalink; N: number of patients in the total study population; n: number of patients with ≥3 preventive medication class cessations on 1 January 2020 in the group.

*On 1 January 2020
